# Unifying Different Cancer Theories in a Unique Tumour Model: Chronic Inflammation and Deaminases as Meeting Points

**DOI:** 10.3390/ijms23158720

**Published:** 2022-08-05

**Authors:** Pablo Hernández-Camarero, Elena López-Ruiz, Juan Antonio Marchal, Macarena Perán

**Affiliations:** 1 Department of Health Sciences, Campus de las Lagunillas SN, University of Jaén, E-23071 Jaén, Spain; 2 Excellence Research Unit “Modeling Nature” (MNat), University of Granada, E-18016 Granada, Spain; 3 Instituto de Investigación Biosanitaria IBS.GRANADA, E-18071 Granada, Spain; 4 Department of Human Anatomy & Embryology, Faculty of Medicine, University of Granada, Avda. de la Investigación 11, E-18016 Granada, Spain

**Keywords:** deaminases dysregulation, AID, APOBEC, ADAR, cancer phenotype plasticity, cancer stem cells, tumour development model

## Abstract

The increase in cancer incidences shows that there is a need to better understand tumour heterogeneity to achieve efficient treatments. Interestingly, there are several common features among almost all types of cancers, with chronic inflammation induction and deaminase dysfunctions singled out. Deaminases are a family of enzymes with nucleotide-editing capacity, which are classified into two main groups: DNA-based and RNA-based. Remarkably, a close relationship between inflammation and the dysregulation of these molecules has been widely documented, which may explain the characteristic intratumor heterogeneity, both at DNA and transcriptional levels. Indeed, heterogeneity in cancer makes it difficult to establish a unique tumour progression model. Currently, there are three main cancer models—stochastic, hierarchic, and dynamic—although there is no consensus on which one better resembles cancer biology because they are usually overly simplified. Here, to accurately explain tumour progression, we propose interactions among chronic inflammation, deaminases dysregulation, intratumor genetic heterogeneity, cancer phenotypic plasticity, and even the previously proposed appearance of cancer stem-like cell populations in the edges of advanced solid tumour masses (instead of being the cells of origin of primary malignancies). The new tumour development model proposed in this study does not contradict previously accepted models and it may open up a window to interesting therapeutic approaches.

## 1. Introduction

It is a well-known fact that genome- and transcriptome-based modifications can greatly influence cellular behaviour. In this sense, two main families of enzymes with nucleotide-editing properties have been described: cytidine and adenosine deaminases. On the one hand, cytidine deaminase catalyses the cytosine-to-uracil conversion; activation-induced cytidine deaminase (AID) is a representative member with single-stranded DNA-editing properties and is mainly expressed in B lymphocytes in physiological conditions. It plays a key role in antibody diversification through “somatic hypermutation” and “class switch recombination” processes [1]. The apolipoprotein B mRNA editing catalytic polypeptide-like (APOBEC) family also belongs to the cytosine deamination group and consists of 11 enzymes with both single-stranded DNA- and RNA editing capacities [2]. On the other hand, adenosine deaminases acting on RNA (ADARs) represent a group of enzymes with adenosine deaminase activity that mediate the adenosine-to-inosine transition and promote the post-transcriptional editing of RNA-based molecules. In mammals, three ADAR family members are described: ADAR1, which mainly acts in repetitive, non-coding RNA sequences, such as Alu motifs; ADAR2, which mainly edits non-repetitive, coding RNA sequences; and ADAR3, which exerts RNA-editing inhibitory roles [3]. Furthermore, alternative splicing of the *ADAR1* gene leads to the generation of the ADAR1p110 nuclear isoform and the main cytoplasmic one (the ADAR1p150 isoform) [4].

In the majority of cases, cancer remains an incurable and deadly disorder, with increasing incidence each year [5]. Notably, the relevance of deaminases in cancer biology, including AID [6], APOBECs [7], and ADARs has been demonstrated [8]. Moreover, it has been confirmed that inflammation is one of the hallmarks of almost all types of cancers [9], many of which remarkably emerge in the background of chronic inflammation [10]. Interestingly, it has been shown that there is a close relationship between pro-inflammatory signals and the upregulation of AID [11], APOBECs [12], and the ADAR1p150 isoform [13], suggesting an important link between inflammation, deaminase activity, and cancer biology.

Another common feature of cancer is its heterogeneity, not only between different tumour types but between different patients with the same malignancies and even the same tumour masses. Regarding this, cancer stem cells (CSCs) have been identified as a particular subpopulation of cancerous cells with dynamic stem-like phenotypes that can be acquired by well-differentiated cancerous cells [14]. Notably, ADAR-dependent RNA editing can induce a significant variety of cellular phenotypes from a limited set of genes. Moreover, ADARs have been observed to play a key role in the acquisition of a CSC-like phenotype by well-differentiated tumour cells [15], suggesting the relevance of non-genetic alterations in the phenotypic changes undergone by malignant cells during tumour progression.

To date, three main tumour development models have been generally accepted by the scientific community: (i) the stochastic model [16]; (ii) the hierarchic model [17]; and (iii) the dynamic theory [18]. Recently, based on the dynamic and changing features of cancer development, we suggested that CSCs may appear in an advanced stage of the primary tumour progression instead of being the cells of origin [19]. In this regard, chronic inflammation and nucleotide-editing driven by deaminases may be crucial mediators of tumour development and growth. Indeed, here we propose the integration of three models along with deaminases to better explain cancer biology: (i) the stochastic theory with the ability of DNA-based deaminases to create genetic heterogeneity; (ii) the dynamic theory and CSC appearances in an advanced tumour stage; and (iii) the hierarchic model and metastatic growth; both (ii) and (iii) are linked with cancer phenotypic plasticity and non-genetic alterations, such as RNA editing. In addition, the roles played by chronic inflammation and deaminases in cancer behaviours are discussed with the goal of suggesting novel therapeutic approaches.

## 2. Deaminases and Cancer Inflammation

It has been widely reported that chronic inflammation is one of the hallmarks of solid tumours, promoting tumour progression and even the appearance of metastasis [9]. Indeed, it is a well-known fact that many cancers emerge in the background of chronic inflammation, which constitutes a risk factor in tumorigenesis [10]. Importantly, the induction of AID expression has been associated with chronic inflammation and oncogenesis [20]. In fact, it has been noted that both the TNFα pro-inflammatory factor and NFKβ pro-inflammatory signalling promoted AID upregulation [11]. Moreover, the dysregulation of AID has been documented to play a relevant role in the progression of lymphoid carcinomas, such as Burkitt’s B-cell lymphoma [6], and non-lymphoid malignancies, such as gastric cancer [21], colorectal cancer [22], and cholangiocarcinoma [11]. In the same regard, a close relationship between inflammatory-related cytokines, such as IFN, TNFα, IL1, and IL6, and APOBEC3s subfamily member expressions have been shown [12]. For example, the correlation between the IFNγ-derived signature and APOBEC3B and APOBEC3C expression has been characterised [23]. Additionally, the positive feedback loop between APOBEC3B and IL6 has been highlighted as a mechanism by which chronic inflammation may be supported in hepatocellular carcinoma through the constitutive activation of the JAK1/STAT3 pathway [24]. The induction of the APOBEC3B expression by canonical and non-canonical pro-inflammatory NFKβ pathways has also been shown [25]. Similarly, the enhancement of APOBEC3A expression by pro-inflammatory factors, including IFNα, IFNγ, or LPs, has also been described [26,27]. Interestingly, it has been observed that APOBEC3A and APOBEC3B cytidine deaminases are the two members of this family of proteins that are the most frequently overexpressed in many types of malignancies [28]. Notably, some studies have revealed that APOBEC3A exhibits the highest enzymatic activity compared to other family components [7], suggesting the special importance of this molecule in cancer biology. In fact, recent studies have exposed the importance of APOBEC3A, not only in cancerous cells but also in stromal cells of the tumour microenvironment (TME). Specifically, it has been shown that APOBEC3A induces the polarization of monocytes/macrophages, including tumour-associated macrophages (TAMs), toward a pro-inflammatory M1-like phenotype through its RNA editing activity [27].

Regarding ADARs, a general increase in the A-to-I RNA editing activity, associated with clinical prognostic values, has been documented in the majority of cancers [8]. Remarkably, it has been demonstrated that the JAK/STAT3 pro-inflammatory pathway promotes A-to-I RNA editing in leukemic stem cells [29]. Strikingly, ADAR1 has also been shown to be a negative regulator of IFN-derived signalling. For instance, it has been shown that ADAR1 induces the upregulation of miR-302a. This event promotes the inhibition of the IRF9/STAT1 pathway and, thus, suppresses the pro-inflammatory signal derived from IFN stimulation in gastric cancer cells [30]. Similarly, it has been reported that ADAR1, apart from the IFN-related response, was able to block the TNFα-derived pro-inflammatory pathway [31]. According to these facts, the inhibition of pro-inflammatory signalling by ADAR1 could be considered a negative feedback loop to control the inflammatory response and avoid chronic ADAR1 overexpression. Furthermore, it is relevant to point out that the nuclear ADAR1p110 isoform exhibits a constitutive expression, whereas cytoplasmic ADAR1p150 expression is inducible by pro-inflammatory signals, such as interferon [13], the latter being of special emphasis in the present article. Interestingly, the importance of the upregulation of the ADAR1p150 isoform, but not ADAR1p110, in the progression, aggressiveness, and growth of several malignancies, such as acute myeloid leukaemia [15], triple-negative breast cancer [32], and melanoma [33] has been revealed, suggesting that chronic inflammation in cancer disease may bypass the cellular control of ADAR1 through the mentioned negative feedback loop. Additionally, some data suggest that ADAR1 could also play an important role in the TME establishment. According to its anti-inflammatory activity, it has been observed that ADAR1 overexpression in macrophages induces the polarization toward an M2-like anti-inflammatory phenotype through the ADAR1/miR-21/FOXO1/IL10 axis [34]. Considering this fact, it seems reasonable to propose that there is a relevant role played by ADAR1 in the TAM M2 polarization characteristics of many solid malignancies, such as triple-negative breast cancer [35]. Consistent with the potential role of ADAR1 in the TME establishment, the expression of ADAR1 in cancer-associated fibroblasts (CAFs) has also been studied, correlating with the expression of CAF-related biomarkers, such as FAPα, and with the abundance of CAFs in colorectal cancer [36]. Indeed, the authors demonstrated that healthy fibroblasts co-cultured with a conditioned medium from colorectal cancer cells overexpressed ADAR1 and increased their invasive potential through AZIN1 mRNA editing, reinforcing the idea about a potential association between ADAR activity and the “malignification” of stromal cells triggered by cancerous cells. To note, previous studies exposed the existence of two main areas within the TME in advanced stages of tumour progression, the pro-inflammatory TME with M1-like TAMs and the anti-inflammatory/regenerative TME with M2-like TAMs (reviewed in [37]).

**Figure 1:**
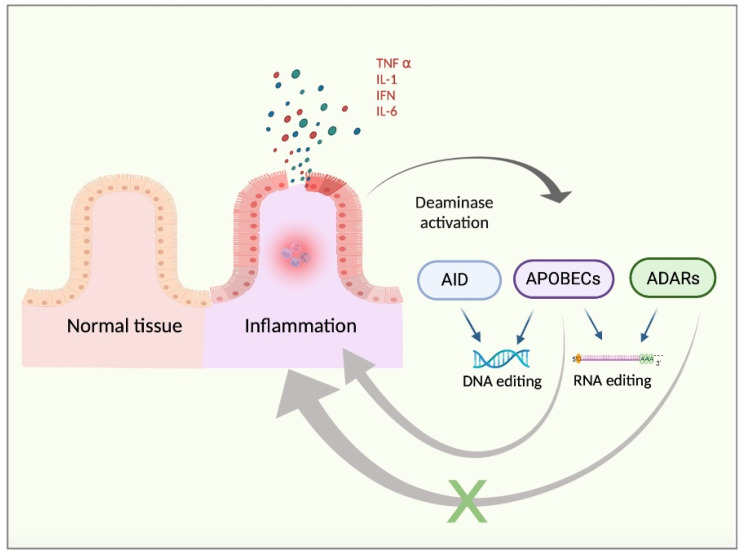
Chronic inflammation background. Inflamed tissue releases pro-inflammatory cytokines, such as TNFα, IFN, IL1, or IL6. These factors may trigger the activation of the pro-inflammatory pathways in the target cells, with NFKβ signalling being a representative example. Such a pro-inflammatory response may lead to increased DNA- and RNA-based editing by enhanced activity of deaminases, such as AID, APOBECs, and ADARs. Moreover, deaminases exhibit opposite regulatory feedbacks in the context of inflammation. To note, APOBEC activity may sustain chronic inflammation in a positive feedback loop whereas ADAR activity may be prone to inducing an anti-inflammatory response, thus exerting negative control feedback.

Therefore, it seems reasonable to propose a link between both i) APOBECs and the pro-inflammatory TME and ii) ADAR1 and the anti-inflammatory/regenerative-like TME, concluding that chronic inflammation may support deaminase implications in cancer progression and TME development. In summary, the expression of deaminases is enhanced by pro-inflammatory signals. Nevertheless, APOBEC activity may further support inflammation in positive feedback, whereas ADAR activity may inhibit the inflammatory responses through a negative feedback loop (Figure 1).

## 3. RNA Editing, Cellular Transcriptome, and Cancer Stem Cells

The role of CSCs in cancer progression is widely accepted, yet doubts remain as to whether they should be considered induced or inducers. Notably, the plastic and changing nature of the CSC-like phenotype, which can be acquired by well-differentiated cancerous cells, has been highlighted [14]. Moreover, it has been proposed that the CSC population may rise from non-stem cancerous cells undergoing the epithelial-to-mesenchymal transition (EMT) process in an advanced stage of primary tumour progression [19]. Interestingly, it has been documented that ADAR1 plays a key role in the generation of induced pluripotent stem cells (iPSCs), promoting the somatic cell plasticity required for the reprogramming process [38]. Furthermore, Crews and colleagues characterised CSC-specific RNA editing events with the prognostic value in leukaemia [39], reinforcing the relationship between ADAR-dependent RNA editing and CSCs biology. Remarkably, a recent study has presented the relevance of ADAR1-dependent RNA editing, induced by pro-inflammatory signals, in the evolution of pre-leukemic stem cells to leukemic CSCs [15]. Interestingly, the link between ADARs and CSCs may be reinforced by the previously suggested close relationship between the appearance of CSCs within a tumour mass and the generation of an anti-inflammatory TME [37]. In addition, ADAR1 action in EMT has also been shown. For instance, it has been observed that both ADAR1 p110 and p150 isoforms exhibit important roles in the EMT triggered by TGFβ supply in oral squamous cell carcinoma. Additionally, the overexpression of ADAR1 enhanced cell migration, invasiveness, and proliferation, and is positively correlated with lymph node metastases, tumour progression, and poor prognosis [40]. Similarly, ADAR1 enhanced proliferation, migration, EMT, and the invasiveness of pancreatic ductal adenocarcinoma through the circNEIL3/miR-432/ADAR1/GLI1-editing axis and, simultaneously, the overexpression of ADAR1 correlated with tumour progression and metastasis [41]. Further, the enhancement of ADAR-dependent RNA editing activity (especially the ADAR1p150 isoform) was noted to promote EMT, cell proliferation, invasion, migration, and even the evolution of thyroid carcinoma toward a more undifferentiated and aggressive type [42]. This study particularly documented the importance of miR-200b editing in the oncogenic role of ADAR1, revealing the potential of altering the transcriptome by RNA editing in the regulation of cancer cell behaviour.

In this respect, the deep impact on the global transcriptome of A-to-I RNA editing, which can alter the RNA structure, stability, subcellular location, coding sequence, binding affinity, and even RNA splicing has been revealed [43]. In addition, the regulatory potential of RNA-based deaminases extends beyond their editing activities, with a good example being the negative regulation of ITGB3 expression in melanoma, both transcriptionally and post-transcriptionally by ADAR1 through an RNA editing-independent mechanism [44]. Focusing on RNA editing activity, a recent study identified several A-to-I editing events during human pluripotent stem cell-to-cardiomyocyte differentiation. Specifically, a minor (but significant) proportion of them were located within transcript coding sequences leading to amino acid changes [45], which may contribute to the generation of new proteins with distinct functions. In parallel with this, it has been reported that A-to-I editing within the mRNA 3′-untranslated region may represent a frequent event in many malignancies and can modify miRNA-binding motifs altering the miRNA putative targets. As an example, the editing of the apoptosis-inducing gene *DFFA* made it targetable by miR-140-3p in some breast cancer cells, leading to specific phenotypic characteristics [46]. A similar effect can be observed when ADAR-dependent editing occurs within the miRNA, which may change its specificity to target different mRNAs [47]. In the context of miRNAs, ADAR1 can also modulate their biogenesis and, thus, can differentially regulate the gene transcripts along with cellular behaviour. A recent study revealed different and specific miRNA signatures in healthy colon cells, non-stem colorectal carcinoma cells, colorectal CSCs, and colorectal carcinoma patients according to the tumour progression stage, including the metastatic phase [48]. For instance, it has been shown that ADAR1 physically interacts with miR-21 precursors, promoting its downregulation in macrophages, which leads to a radical change in their phenotypic polarization [34]. Moreover, ADAR1 can influence the ratio of non-coding RNAs, such as miRNAs, processed through an RNA editing independent manner, by forming a complex with miRNA biogenesis-related factors, such as Dicer [49]. Furthermore, gene transcripts can be regulated by alternative splicing to generate different protein isoforms. Remarkably, it has been reported that splicing-related motifs frequently harbour A-to-I editing sites [50]. On the other hand, ADAR1-dependent editing activity can also influence alternative splicing through a different mechanism relying on the editing of splicing-associated factors. For example, Ramírez-Moya and co-workers documented the ADAR1-related editing of the CDK13 transcript, a splicing regulator in thyroid cancer, which alters the splicing patterns and promotes cell viability, proliferation, and invasiveness [51]. Notably, splicing patterns conditioning the RAC1/RAC1b transcriptional ratio have been presented as a key factor in the maintenance of the pancreatic CSCs phenotype [52].

**Figure 2:**
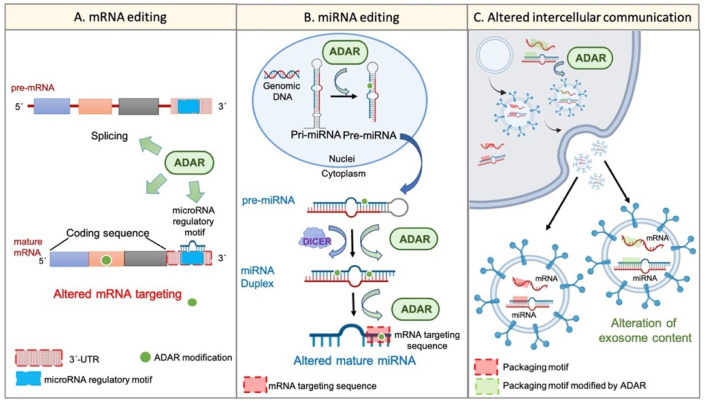
Wide transcriptome alteration by ADARs. Panel (**A**) shows the ADAR-mediated mRNA editing. ADARs can influence pre-mRNA maturation by interfering with the splicing process. Furthermore, ADARs can edit mature transcripts on different sites. For instance, ADARs can modify the “miRNA regulatory motif” within the 3′UTR region and, thus, can alter mRNA regulation by certain miRNAs. On the other hand, ADARs are able to edit the coding sequence of the mature transcript, thereby conditioning the final protein. Panel (**B**) represents the miRNA alteration by RNA editing. ADARs can regulate the multistep biogenesis of miRNAs in different ways; for example, the direct precursor editing or the physical interaction with regulatory factors, such as Dicer. Additionally, ADARs may edit the mature miRNA within its “mRNA targeting sequence”, thereby altering its mRNA targeting potential. Panel (**C**) shows the potential regulation of intercellular communication and extracellular vesicle cargos by single-nucleotide editing. RNA editing by ADARs can modify specific sequences within cellular mRNAs and miRNAs, such as the “zip code-like sequence” or the “surface targeting motif”, respectively (“packaging motifs”). The editing of such motifs may condition the packaging of certain mRNAs and miRNAs into extracellular vesicles, such as exosomes, and may alter the intercellular communication and the coordinated behaviour of a cellular population.

On the other hand, the potential of exosome content as a prognostic tool in cancer disease has been proposed. It was suggested that exosome cargos (including RNA-based molecules, such as mRNA or miRNAs) vary according to the tumour progression stage, tumour aggressiveness, invasiveness, metastatic capacity, or drug resistance [53]. A similar event was described regarding TME-derived vesicles, such as CAF-derived exosomes, whose cargo miRNAs differed from that of healthy fibroblast-derived exosomes [54]. Remarkably, the existence of specific sequence motifs within miRNA, named “surface targeting motifs”, which may be implicated in the miRNA packaging into extracellular vesicles, including exosomes, has been described [55]. Additionally, the presence of a 25-nucleotide zip code-like sequence element in the 3′UTR of mRNA transcripts responsible for the mRNAs packaging into exosomes [56] has also been described. Considering the potential of ADAR-dependent RNA editing to modify RNA sequences, it seems reasonable to suggest that the dysregulation of ADAR activity may also explain, at least partially, the changes in cellular behaviour, transcriptome, phenotype, and even exosome cargos and intercellular communication during tumour progression and TME generation (Figure 2).

In addition to ADAR-dependent RNA editing, other deaminases of the APOBEC family, such as APOBEC3A [57], exhibit RNA editing activity and, thus, may also have the potential to induce relevant transcriptome changes in cancer disease. Moreover, the potential of RNA-based deaminases and RNA editing to promote significant phenotypic changes may be enhanced by the existence of other RNA-based molecules, also susceptible to being edited, with a relevant role in CSC maintenance, for example, circular RNAs [58] or long non-coding RNAs [59]. Taking all of this into account, these data suggest that RNA editing may provide significant transcriptome diversity from a limited source of a gene set, subsequently leading to proteomic diversity and cancer progression, towards a more aggressive and invasive phenotype, such as the CSC-like one [60]. Nonetheless, to the best of our knowledge, there is no evidence of a direct causative relationship between RNA editing and the acquisition of a stem-like phenotype by cancerous cells, so this idea remains theoretical, which may prove interesting to clarify in future studies.

## 4. Deaminases and Our Understanding of Tumour Progression

It is a well-known fact that the extreme heterogeneity of cancer makes it difficult to reach a consensus on a unique tumour development model. Although three main models have been generally accepted based on stochastic, hierarchic, and dynamic theories, there is still some controversy regarding the matter. The stochastic model relies on the “Clonal Evolution hypothesis”, which postulates that intratumor genetic heterogeneity, shown as subclonal differences, is a consequence of genomic instability and accumulation of the variable mutational burden during each cellular replication. Although this theory may explain intratumor genetic heterogeneity, it evades the existence of phenotypic/functional diversity, such as the highly-differentiated and stem-like phenotypes, between genetically similar subclones [16]. Focusing on the different intratumor genetic variants, it may be relevant to highlight the potential of DNA-based deaminases to induce single-nucleotide genetic alterations. In fact, distinctive APOBEC-mutational spectra have been observed in many types of malignancies [61]. Similarly, Ye and co-workers described AID-specific mutational patterns in certain subtypes of lymphomas [62]. Thus, deaminases acting on DNA strongly contribute to the generation of genetically distinct subclonal populations within a tumour. Examples include, among others, APOBEC3B in breast cancer [63] or AID in B cell lymphoma [64]. Furthermore, changes in the deaminase-associated mutational signatures during tumour progression have been identified and proposed as a model of tumour growth [65]. To note, both AID [64] and APOBEC [66] hyperactivity have also been related to tumorigenesis, suggesting a crucial role played by aberrant DNA-editing as the start point of cancer growth.

On the other hand, the hierarchic model proposes a hierarchical organization within the tumour mass, with CSCs representing the top of the hierarchy. This theory states that tumorigenesis starts when a healthy stem cell escapes from proliferation control due to the accumulation of a specific mutational burden and becomes a CSC. Next, this CSC will generate the intratumor heterogeneity and the distinct subpopulations of well-differentiated cancerous cells through asymmetric divisions [17]. Nonetheless, this model ignores the documented intratumor subclonal genetic diversity within a single tumour mass. Similar to the hierarchic theory, the dynamic model focuses on the intratumor phenotypic heterogeneity but the CSC-like phenotype may not be stable over time. Conversely, the stem-like features could be acquired by well-differentiated cancerous cells through specific environmental cues leading to the generation of new CSCs [18]. Consistently, it seems reasonable to state that such phenotypic flexibility could be associated, at least partially, with non-genetic alterations, such as RNA editing (see previous sections), and more, considering that it has been shown that ADAR-dependent A-to-I RNA editing could mediate the upregulation of key oncogenes and the downregulation of important tumour suppressor ones [67]. In the same regard, it has been shown that AID can induce epigenetic modifications, functioning as DNA demethylation [68]. Interestingly, the AID demethylating activity, rather than its mutagenic potential, has been associated with the enhancement of proliferation, invasiveness, migration, metastatic capacity, and the promotion of the EMT process in renal cell carcinoma [69]. Similar results were described by Li’s group, which reported that AID promoted cell proliferation, migration, and invasiveness in bladder cancer through DNA demethylation [70]. In accordance with this, it has been previously noted that metastatic processes (presumably driven by CSCs) may not rely on metastasis-specific mutations but epigenetic alterations [71].

Furthermore, as it has been proposed, reflecting on the beginning of what one usually knows as “advanced stage of tumour progression”, CSCs may come from well-differentiated cells located in the tumour mass edges instead of being the cells of origin of the primary tumour (CSCs may be induced during primary tumour development rather than being the inducing cells) [19]. According to this hypothesis, ADAR-dependent editing activity, in an advanced stage of tumour progression may contribute to promoting a deep phenotypic switch leading to the appearance of CSCs, thus, showing the relevance of post-transcriptional RNA editing in advanced stages rather than in an early phase of primary tumour progression. In agreement with this, it has been observed that the ADAR-dependent editing of CSF3R, MSH2, NUMA1, and KDM2 was only observed in metastatic lesions of lung cancer but not in primary tumours [72]. Furthermore, a recent study noted the importance of both DNA- and RNA editing in the progression of acute myeloid leukaemia. It was established the mutagenesis driven by APOBEC3 family members was a “pre-requisite” prior to the phenotypic changes driven by ADAR RNA editing [15]. Importantly, RNA editing may not be the only factor contributing to such a phenotypic switch, with metabolic changes being another representative example [73], but it may still play a central role. Additionally, other studies have pointed out the importance of the combination of both mutagenesis and environmental cues in cancer biology. For instance, it has been demonstrated that the mutagenesis in the *KRAS* gene on its own was not sufficient to produce pancreatic ductal adenocarcinoma, and that epigenetic alterations, i.e. changes in DNA methylation patterns, derived from pro-inflammatory/tissue damage-associated signalling were also required [74]. Interestingly, non-genetic changes, such as RNA editing, generally exhibit a transitory nature, considering the short half-life of RNA-based molecules [75], reinforcing the relevance of chronic rather than acute or punctual inflammation in carcinogenesis and tumour progression in order to induce lasting phenotypic changes derived from transcriptome-based modifications.

**Figure 3:**
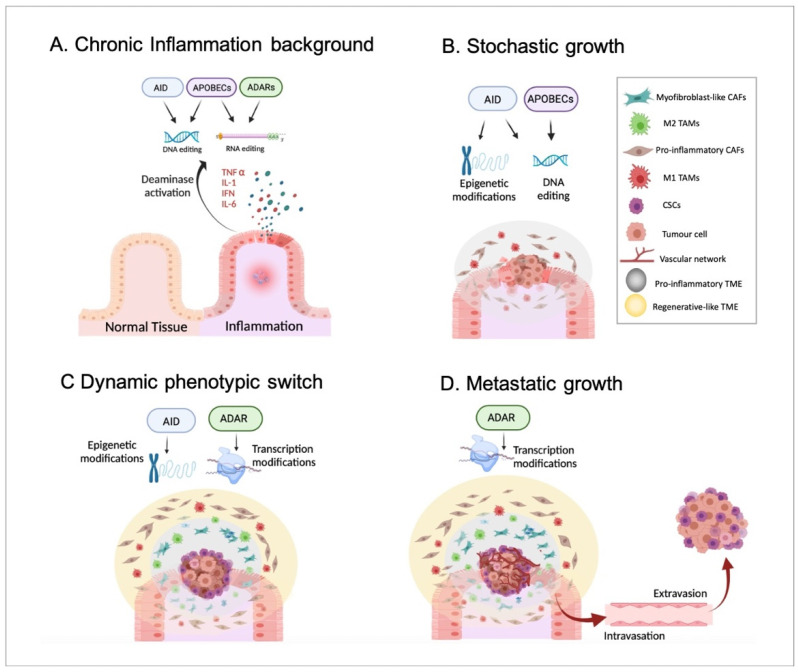
Inflammation, deaminases, and tumour progression. In Panel (**A**), a background of chronic and non-resolved inflammation that may alter the activity of both DNA- and RNA-based deaminases, including AID, APOBECs, and ADARs is presented. In Panel (**B**), the emergence and stochastic growth of the primary tumour in the earliest stages are shown. The collaboration of both epigenetic and genetic alterations driven by DNA-based deaminases in the emergence of malignant cells is highlighted. Moreover, DNA-editing may induce the appearance of genetically distinct cancerous cell subpopulations according to the stochastic model. Additionally, a pro-inflammatory TME with pro-inflammatory CAFs and M1 TAMs is generated and sustained by, at least partially, APOBEC activity. Panel (**C**) identifies the appearance of CSCs on the edges of the tumour mass in an advanced stage. Non-genetic alterations, such as epigenetic modifications and constitutive RNA editing, may allow a phenotypic switch underlined by the acquisition of a CSC-like phenotype by non-stem cancerous cells located mainly in the borders. Furthermore, the appearance of CSCs may induce the generation of an anti-inflammatory/regenerative TME, with myoCAFs and M2 TAMs, just adjacent to CSCs. In such events, ADARs may play a relevant role by promoting an anti-inflammatory response. Panel (**D**) shows the final stages of tumour progression in which CSCs may migrate (through intravasation and extravasation) and generate a metastatic tumour with a hierarchical organization. It seems reasonable to assume that there is an important role played by ADAR RNA editing in the phenotypic switch during the differentiation of CSCs into well-differentiated cancer cell subpopulations.

Indeed, a pro-inflammatory TME has been suggested to be generated by tumour cells from the earliest stages of tumour growth and chronically maintained during cancer progression, prior to the appearance of an anti-inflammatory/regenerative TME in the advanced stages [37]. Importantly, the pro-inflammatory environment followed by an anti-inflammatory/regenerative one may be in agreement with the aforementioned APOBEC-derived DNA-editing prior to ADAR-dependent phenotypic changes (according to everything presented in Section 2). To summarise, inflammatory disorders, including viral infection-driven inflammation [66], may promote the generation of genetic mutations produced by the DNA-based deaminases, such as APOBECs or AID and, thus, cellular malignization. In parallel with this, epigenetic alterations, for example, changes in methylation patterns driven by AID, might also contribute to tumorigenesis. Together, these events driven by inflammatory signals may promote the emergence of a primary tumour, which may trigger the formation of a pro-inflammatory TME. Next, such a pro-inflammatory environment may support the stochastic growth of the primary tumour, highlighting the generation of the characteristic intratumor genetic heterogeneity and different subclonal populations due to, at least in part, the constitutive activity of DNA-based deaminases. Then, in a more advanced stage, the inflammatory environment chronically maintained during tumour growth may induce well-differentiated cancer cells located in the tumour mass border to become CSCs. This phenotypic switch may mainly be caused by non-genetic modifications, such as AID-dependent demethylations and ADAR-mediated constitutive RNA editing. As previously mentioned, cancerous cells may require sufficient time to accumulate specific mutations until the phenotypic switch can be completed. In other words, the acquisition of such specific mutational burdens may allow malignant cells to react differently to external environmental signals and become CSCs. Obviously, this idea requires further confirming experimental studies. Moreover, such a CSC population may promote the generation of an anti-inflammatory/regenerative TME adjacent to the tumour mass. Finally, CSCs located in the tumour edges would trigger the formation of distant metastatic tumours in the last stages of cancer progression. To note, the hierarchical growth of metastatic tumours and the differentiation of CSCs into distinct cancer subpopulations may also rely on phenotypic changes and, thus, on non-genetic events, including RNA editing. Therefore, it seems reasonable to propose a crucial role of ADARs in such metastatic growth (Figure 3).

Regarding the huge heterogeneity of cancer, it is true that not all human malignancies may emerge from a background of chronic inflammation. Nevertheless, deaminase dysregulation could also be driven by other factors. As an example, the dysregulation of APOBEC3C through gene mutations and the expression alterations mediated by non-coding RNAs have been associated with breast cancer risk and progression [76]. Thus, the model presented here could be applied, at least in part, to the absence of an inflammatory background considering that one of its main pillars relies on deaminase alterations.

Reinforcing the possibility of considering DNA-based deaminase dysfunctions as the start point of tumour development may need additional research since, putting AID aside, it has been suggested that APOBEC aberrant activity may not be detected at an early stage of cancer progression in all types of malignancies [23]. Another consideration relies on the presence of an inflammatory microenvironment from the earliest stages of tumour progression, which makes it reasonable to assume that both APOBEC- and ADAR-derived signalling may be simultaneously active in tumour cells. Therefore, the previously mentioned APOBEC-positive feedback around inflammation and the ADAR-negative one may represent an “antagonizing co-existence”. As far as we are concerned, currently, there is no information comparing the relative strengths of both regulatory feedbacks, but one could throw some light on this unknown area. In the early stages of tumour development, the APOBEC-positive feedback around inflammation may predominate over the ADAR-negative one, whereas the accumulation of additional specific mutations and/or the appearance of CSCs in later stages may contribute to a switch in the said balance, promoting the predominance of anti-inflammatory signalling. In this context, the activity of DNA-based deaminases, such as APOBECs, may exist in all stages of the tumour progression timeline instead of only being present in earlier phases. In support of this, it has been documented that metastatic tumours may exhibit a higher mutational burden compared to primary ones [77], although it does not necessarily imply that such additional mutations are the drivers of metastatic spread. However, it is possible that specific DNA mutations could promote the acquisition of a CSC-like phenotype by well-differentiated cancerous cells, so it may be important to design future studies focused on determining how frequent such an event is within human cancers. Moreover, although it has been shown that the majority of tumours usually present a significantly elevated A-to-I RNA editing activity [8], the promotion of ADAR1 activity does not correlate with tumour progression and poor prognosis in all these malignancies. For instance, it has been revealed that the downregulation of ADAR1 strongly correlated with cancer invasion, progression, and metastasis in melanoma [44]. Therefore, it could be interesting to research further the relationship between inflammation, ADARs, and RNA editing in this malignancy type, for instance by separately studying ADAR1p110 and ADAR1p150 isoforms. Regarding inflammation, one could consider that the generation of a pro-inflammatory TME with M1 TAMs by an individual’s tumour cells from the earliest stages of cancer development may be a contradictory event according to the anti-cancer behaviour of M1-like TAMs presented above. Nonetheless, the upregulation of CD47 by cancerous cells has been identified and this allows them to strongly mitigate their targeting by immune cells, such as macrophages [78]. In other words, malignant cells may be able to evade anti-cancer M1-like TAMs while indirectly benefiting from a pro-inflammatory environment. Furthermore, the opposite TAM polarization towards an anti-inflammatory, tumour-supportive M2-like phenotype in later stages may represent direct support for tumour cells by an inflammation-independent mechanism. In agreement, it has been documented that M2 macrophages enhanced proliferation, invasion, migration, tumour formation, the EMT process, and metastasis in colon cancer cells [79].

## 5. Diagnostic and Therapeutic Considerations

A significant proportion of cancer mutational burden may be environmentally-dependent, having an intrinsic order rather than exhibiting a random nature associated with inherent replication mistakes. In fact, it has been noted that a significant proportion of single-nucleotide alterations in many types of malignancies, such as bladder, head and neck, breast, cervix, and lung cancer can be classified as APOBEC-specific mutational patterns [63]. Remarkably, unlike random events, causative events driven by well-defined factors and molecules may have, at least partially, “predictable” and “controllable” sides. Regarding diagnosis, an association between the overexpression of APOBEC3A and/or APOBEC3B and certain clinical features in breast cancer, including the absence of ER, PGR, and HER2 receptors, high histological grades, activation of proliferation-related gene sets, and poor clinical outcomes, has been determined. Conversely, APOBEC3C-H expression patterns correlated with pro-inflammatory-related gene sets, a specific tumour immune landscape, and a better prognosis [63]. Moreover, the authors associated the overexpression of APOBEC3A-B with other genetic aberrations, including neoantigen copy number alteration, aneuploidy, or homologous recombination defects. Delving deeper, focusing on the molecular entities responsible for these genetic alterations could provide additional valuable information. A good example could be the prediction of the most probable locations of single-nucleotide mutations based on the overexpression of a specific APOBEC family member, which may greatly facilitate the diagnostic process. Regarding this idea, it has been observed that the APOBEC-mediated deamination preferentially occurs in TCW-specific DNA sequence motifs (“W” being any type of nucleotide) [80]. Furthermore, structural studies revealed substrate specificity differences between distinct members of the APOBEC3 group, such as APOBEC3A, 3B, or 3G [28], showing the existence of deamination motif heterogeneity regarding each deaminase. Therefore, an in-depth analysis of solid tumour biopsies could be proposed to establish a correlation between the activity of specific deaminases and the most probable mutational genome map. This information could provide an approximate idea about the most probable evolutionary path regarding the acquisition of different mutational sets of an early-diagnosed cancer, to predict, at least in part, the best therapeutic line. As an example, an association between certain types of mutations and a better response to anti-cancer drugs (for instance, afatinib or neratinib in the case of cervical cancer) has been revealed [80].

In the context of “controlling” the mutational events, the inhibition of specific APOBEC members that are overexpressed in many cancer types could be proposed [81]. However, it is important to point out that such a strategy would only prevent the acquisition of additional APOBEC-associated mutations, but it would not reverse those already acquired. To this end, the development of different molecular constructs combining the Crispr/Cas9 technology with DNA-based deaminases, such as AID or APOBECs with the objective of obtaining a targetable DNA based-editing tool has been studied [82]. However, there are still key limitations, such as off-target effects or wide editing windows [28]. Hence, a targetable DNA-editing approach seems suitable to be used in pre-clinical studies rather than in clinical practice in the near future.

Interestingly, Wang and co-workers have identified around 19 A-to-I RNA editing hotspots in many types of malignancies, which were associated with tumour stage and patient overall survival [83]. Similarly, the existence of specific RNA modification patterns that correlated with patient overall survival, metastasis, specific signalling pathways, anti-cancer drug response, and even TME composition (i.e., M2 TAM infiltration) in colorectal cancer has also been demonstrated [84]. Similar to DNA-editing factors, it seems reasonable to assume that each RNA editing molecule may exhibit its own site-specific activity. For instance, it is known that ADAR1 mainly acts in repetitive, non-coding RNA sequences, such as Alu motifs, whereas ADAR2 mainly edits non-repetitive, coding RNA sequences [3]. A recent comprehensive and quantitative study documented distinct specific sequences edited by ADAR1, ADAR2, or by a coordinated interplay between the two enzymes [85]. Additionally, it has been pointed out that the majority of RNA editing events belong to A-to-I modifications rather than to C-to-U ones, which, along with the data described earlier, can significantly simplify the RNA editing analysis in clinical practice by using next-generation RNA sequencing techniques [86].

The use of RNA editing factors to induce controlled changes in RNA-based molecules could represent a potential therapeutic approach. As an example, the development of an APOBEC3A mutant, without its DNA-based editing activity, to avoid off-target activities on DNA and, thus, restricting its base editor function only to RNA-based molecules, has been shown [57]. However, controlled RNA editing strategies still present several limitations, such as the lack of efficiency, bystander editing, or sequence restrictions, which need to be addressed before its therapeutic use as an alternative to genome editing [87]. In addition, it must be taken into account that RNA-based molecules exhibit a transient and short half-life only, unlike DNA. This fact may imply that any strategy based on RNA modification should be a long-term and constitutive treatment rather than a single-dose therapy in order to achieve relevant therapeutic effects. Similarly, this idea may be extrapolated to other strategies: silencing upregulated ADARs, direct RNA molecule-related therapies, such as the dysregulated miRNA blockade [88] and/or anti-inflammatory strategies in order to alter RNA editing. An interesting feature of the transient nature of RNA editing may be its reversibility without the long-term maintenance of the triggering signals of such an event. This hypothesis may imply the need for continuous signalling to achieve RNA editing-derived biological effects, including the stable maintenance of the CSC-like phenotype. In other words, without such constitutive signals, the stem-like phenotype would be lost after a short period of time. In support of this idea, the dependence of the CSC-like phenotype on continuous environmental cues, such as low-attachment conditions, has been reported [89].

## 6. Conclusions

To date, cancer is one of the most deadly and scariest diseases worldwide despite the huge efforts made by the scientific community to achieve effective treatments. To this end, a better understanding of tumour progression represents a crucial factor in defining and establishing the best therapeutic targetable bases. Nonetheless, there is no consensus on tumour development as the widely-accepted models seem to be oversimplifications. Focusing on the common features of nearly all types of malignancies, such as chronic inflammation, deaminase dysregulation, or DNA- and RNA-based editing alterations could help us to identify crucial points, which may lead to the proposal of more accurate tumour development models and the significant improvement of its clinical management. Here, we propose a dynamic association of the key features of tumour development, including chronic inflammation, deaminase dysfunctions, intratumor genetic heterogeneity, and cancer phenotypic plasticity. Importantly, the idea presented in this study does not contradict any of the previously established postulates and experimental observations, but it may present a more accurate point of view with interesting clinical implications. In summary, the model proposed here combines previously established tumour development theories: primary tumours may emerge in the background of chronic inflammation and may grow at the earlier stages according to the stochastic hypothesis. In an advanced stage of tumour progression, the CSC population coming from well-differentiated cancer cells may appear at the edges of the solid tumour mass according to the dynamic model. Finally, CSCs may migrate to distant organs where they may promote metastatic growth according to the hierarchic theory.

## Data Availability

Not applicable.

## Abbreviations

ADARs	adenosine deaminases acting on RNA
AID	activation-induced cytidine deaminase
APOBEC	apolipoprotein B mRNA editing catalytic polypeptide-like
CSCs	cancer stem cells
EMT	epithelial-to-mesenchymal transition
iPSCs	induced pluripotent stem cells
CAFs	cancer-associated fibroblasts
TAMs	tumour-associated macrophages
TME	tumour microenvironment
